# Bioremediation of hazardous heavy metals by marine microorganisms: a recent review

**DOI:** 10.1007/s00203-023-03793-5

**Published:** 2024-02-15

**Authors:** Ahmed N. Alabssawy, Amr H. Hashem

**Affiliations:** 1 Marine Science and Fishes Branch, Zoology Department, Faculty of Science, Al-Azhar University, Cairo, 11884 Egypt; 2 Botany and Microbiology Department, Faculty of Science, Al-Azhar University, Nasr City, Cairo, 11884 Egypt

**Keywords:** Heavy metals (HMs), Biological treatment, Hazardous materials, Marine microorganisms, Ecosystem, Bioremediation

## Abstract

Heavy metals (HMs) like Zn, Cu, Pb, Ni, Cd, and Hg, among others, play a role in several environmental problems. The marine environment is polluted by several contaminants, such as HMs. A variety of physico-chemical methods usually available for sanitation HMs remediation suffer from either limitation. Bioremediation is a promising way of dealing with HMs pollution. Microbes have the ability with various potencies to resist HMs tension. The current review discusses the main sources and influences of HMs, the role of marine microorganisms in HMs bioremediation, as well as the microbial mechanisms for HMs detoxification and transformation. This review paper aims to provide an overview of the bioremediation technologies that are currently available for the removal of HMs ions from industrial and urban effluent by aquatic organisms such as bacteria, fungi, and microalgae, particularly those that are isolated from marine areas. The primary goals are to outline various studies and offer helpful information about the most important aspects of the bioelimination techniques. The biotreatment practices have been primarily divided into three techniques based on this topic. They are biosorption, bioaccumulation, bioleaching, and biotransformation. This article gives the brief view on the research studies about bioremediation of HMs using marine microorganisms. The current review also deals with the critical issues and recent studies based on the HMs biodetoxification using aquatic microorganisms.

## Introduction

The natural environment has been contaminated by the ongoing accumulation of heavy metals and metalloids, stemming from the rapid growth of industrial practices, ore mining, and the disposal of high levels of metal waste. Heavy metals pose a significant challenge as pollutants that are not easily broken down. These pollutants can originate from either natural sources or human activities (Tan et al. 2021). The rapid expansion of anthropogenic activities, industrialization, and urbanization brought on by the industrial revolution over the past few decades are thought to pose a serious threat to public health. Direct disposal of toxic heavy metals (HMs) including lead, cadmium, and chromium results in serious ecological issues that have a negative impact on a variety of ecosystems (Fernández et al. 2014). Like a marked increase in ecological load because of water contaminated and shortage required the isolation of HMs from manufacturing polluted water (Luo et al. 2014). These toxic metals have a lasting impact on the environment as they cannot be broken down or destroyed. They have the ability to spread and accumulate throughout the food chain once they are introduced into the environment. Furthermore, they have the ability to infiltrate the human body either through airborne transmission or direct contact with the skin, posing significant risks to human well-being (Ji et al. 2019; Liu et al. 2020). Different mechanisms laid out to assure the effective remediation of polluted waters combat the sequel of the random emission of HMs in many water flats. Accordingly, various remediation techniques, like chemical residue, oxidation/reduction, ion swap, membrane refinement, and vaporization, have been evolved to conquer the aggregation of HMs in polluted areas (Ahmed and Ahmaruzzaman 2016). But traditional processes for remedying fatal HMs in manufacturing junk water are deemed ineffective and costly. Most heavy metals in the environment have poisoning effects, such as carcinogenic, teratogenic, and mutagenic effects, and a certain degree of accumulation (Si et al. 2020). Microorganisms like microalgae, fungi, yeast, and bacteria are known for bioremediation of HMs. Microbes have demonstrated remarkable effectiveness in the remediation of environmental pollutants (Ahmed et al. 2022; Fouda et al. 2015). Their ability to grow quickly and be easily manipulated makes them invaluable in this field (Hashem et al. 2020). It is crucial to enhance the utilization of microbes as a means of bioremediation in order to foster a sustainable environment (Ayilara and Babalola 2023; Hasanin et al. 2020). The utilization of various bioremediation techniques, including biosorption, bio-reduction, bioaccumulation, myco-remediation, bacterial bioremediation, and phytoremediation, are economical and environmentally responsible approaches when it comes to remediation (Singh et al. 2023). Biosorption, the least expensive and environmentally sustainable technique, is employed for the retrieval of HMs ions from water supplies that have been compromised by contamination (Jobby et al. 2018).

**Fig. 1:**
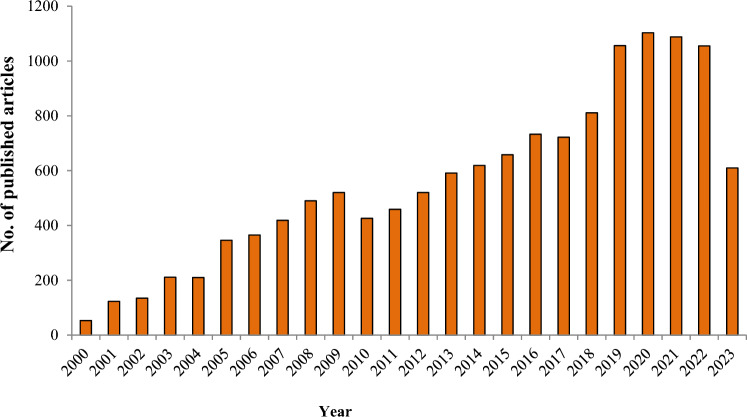
The increase in terms of publications about bioremediation of heavy metals from 2000 to 2023. This statistic was taken from PubMed using “Bioremediation of heavy metals” term

The microbial habitats within marine ecosystems exhibit remarkable specialization and encounter challenging conditions due to the dynamic interplay of factors such as pH fluctuations, precipitation events, salinity gradients, wind dynamics, temperature variations, oceanic currents, and elevated concentrations of HMs in seawater, which can exert deleterious effects on their survival. Henceforth, owing to the intricate nature of environmental conditions, microorganisms exhibit inherent potential for adaptability within this intricate milieu, encompassing adverse circumstances and intricate specific adaptations. Therefore, it is hypothesized that microorganisms obtained from marine habitats possess a greater potential for investigation and application in the process of bioremediation for xenobiotics, hydrocarbons, and HMs. This is attributed to their capacity to generate extracellular polymeric substances/enzymes and synthesize biofilms, as highlighted in the study conducted by Husain et al. (2022). Figure 1 depicts the rise in the number of publications about bioremediation of HMs from 2000 to 2023, where a number of publications are increased gradually in last period, also the number of publications in 2019, 2020, 2021, and 2022 was 1056, 1103, 1088, and 1055 articles, respectively. This indicates the direction of scientists to use microorganisms for solving many current problems especially HMs. Herein, this review aims to discuss dangerous HMs biologically treated by marine microorganisms like bacteria, fungus, and microalgae.

## Heavy metals

**Fig. 2:**
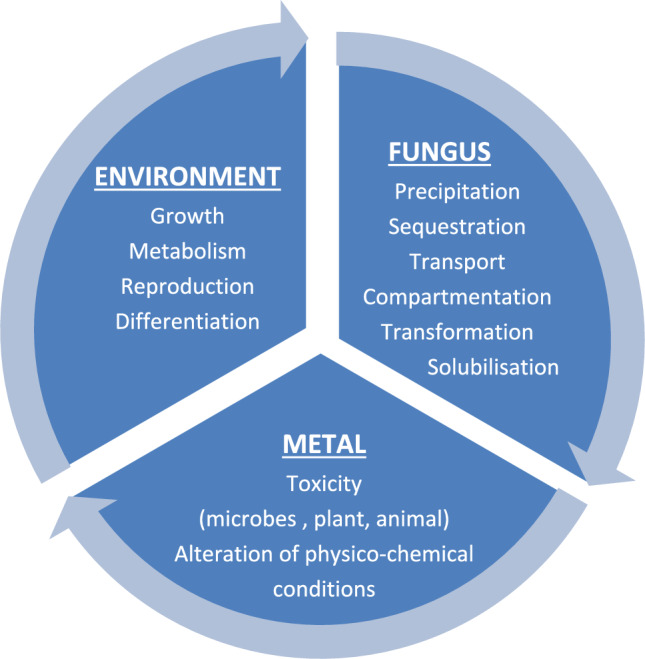
Diagrammatic representation of the dynamic interrelationships between fungi, toxic metals (and related substances), and the environment

There are different levels of toxicity for the organic and inorganic contaminants produced in the effluent. Certain heavy metals play a crucial role in the human biological process. However, excessive intake of these metals can have unexpected and harmful effects on health and the physiological system. Research conducted by Kim et al. (2019) reported that heavy metals have carcinogenic properties, despite their potential health benefits. Various metals can enter the environment as soil, water, and air pollutants. These pollutants can then make their way into the food chain and ultimately affect humans. The presence of these metals can cause significant harm to our cellular system and increase the risk of cancer. Based on the findings of the International Agency for Research on Cancer, it has been determined that certain non-essential heavy metals, such as As, Cd, and Cr, have significant carcinogenic properties. Moreover, organic contaminations are treated using biological, chemical, and physical methods. But these methods are inappropriate for inorganic contaminants like HMs. Due to their qualities such as dissolvable, oxidation–reduction features, and complex construction, HMs breakdown represents an important worry (Lee and Pandey 2012). The substance is HMs if its specific gravity is more than 5.0 and its atomic weight is between 63.5 and 200.6 (Srivastava and Majumder 2008). They appear in the ecosystem as a natural material. The term HMs mentions a substance with great density and poisonous even at a little concentration. Currently, HMs in wastewater are one of the ecosystem’s primary problems, because there is a significant risk to the environment and to human health, even at very low amounts (Fig. 2). HMs pollution is a significant environmental burden because of its adaptability, accumulation, inability to decompose, and stability (Peligro et al. 2016). Industries that discharge HMs into the surroundings include those that produce paper, pesticides, tanneries, numerous metal plating’s, mining processes, etc. HMs have been discovered to be non-biodegradable and harmful to human physiology and other biotic systems. However, they could transform to minimal hazardous material. The higher-poison HMs remain in chemical form or blended form, so it is hard to eliminate from the junk water (Han et al. 2016). Most HMs produce basic compounds which were demanded by biotic organisms in little quantity for metabolic vitality; simultaneously, these HMs have a high potential for harming human health (Koedrith et al. 2013). The disposal of HMs in open water is causing low oxygen levels and algae growth, which is wiping out aquatic life, also results in after discharge into the freshwater, and the HMs get transformed to hydrated molecules that are more poisonous than the metal molecules. These hydrated molecules hold up the enzymes compose operation, also the quick absorption process in it. Hence, proved the elimination of HMs was obligatory to decrease public perils. To lessen the degree of water pollution, international organizations like (WHO) and (EPA) limited the most permissible drainage levels of HMs in the ecosystem. So far, the drainage outflow includes a huge level of HMs larger than admissible levels which lead to hazard problems to all living beings in the environment.

## Heavy metals in the aquatic environment

Over the past few decades, the issue of heavy metal pollution in the marine environment has emerged as a worldwide concern (Romano et al. 2021). In general, the rise in pollutants can be attributed to the lack of comprehensive marine environmental regulations and the growing pollution from human activities such as industry, agriculture, and urbanization. In the Mediterranean Sea, tourism development has emerged as a significant contributor to the pollution of marine areas, impacting the natural environment with the presence of heavy metals (Quevedo et al. 2012; Romano et al. 2021). One of the primary sources of (HMs) pollution is industrial expansion. The “hotspots” for HMs pollution have been the coastal and marine habitats that receive the effluents that are loaded with HMs (Naser 2013b). Also, Sewage is released in large quantities into the coastal and marine ecosystem. Sewage discharges may also contain highly suspended particles of HMs and a substantial amount of nutrients, which can negatively affect life (Naser 2013b; Singh et al. 2004). In addition, activities like dredging and reclamation reduce the marine biota’s abundance, biodiversity, biomass, and richness (Smith and Rule 2001). Further, these activities mobilize higher concentrations of HMs, which allow them to infiltrate food chains ingredients and endanger human health (Guerra et al. 2009). Despite the necessity for freshwater, desalinated seawater is used, particularly in countries of the Arabian Gulf, where minimal precipitation and extreme aridity prevail (Hashim and Hajjaj 2005). Increased levels of HMs have been seen close to desalination plants throughout the Arabian Gulf coastline as a result of the daily release of rejected waters from desalination plants to coastal and subtidal areas (Naser 2013a). Finally, significant amounts of oil contamination are caused by operations related to oil exploration, production, and transportation (Velastegui-Montoya et al. 2023). According to reports, a significant oil spill during the Gulf War in 1991 has resulted in increased levels of HMs (Al-Arfaj and Alam 1993).

## Bioremediation techniques

**Fig. 3:**
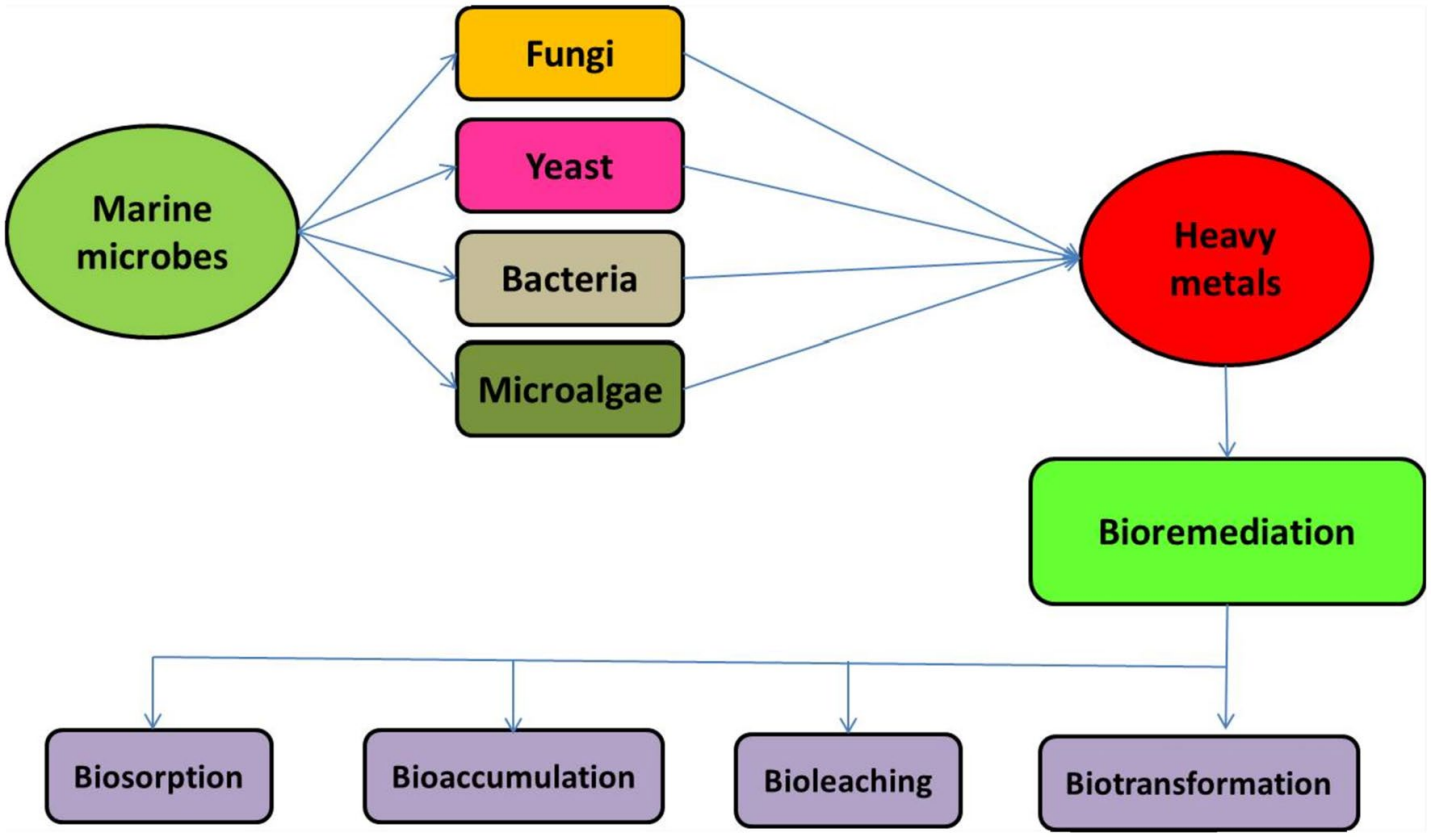
Bioremediation of heavy metals using marine microbes

The method of bioremediation involves removing poisonous substances as well as dangerous toxins from a polluted environment, such as heavy metals, or changing them into less dangerous chemicals (Fig. 3). The process of biodegradation involves the breakdown of biodegradable substances, resulting in the ultimate transformation of organic material into N_2_ gas, CO_2,_ H_2_O, and other by-products. This process can be facilitated by the utilization of biomass, whether in the form of deceased organisms or living organisms and is also applicable in the field of bioremediation. The utilization of both in-site and out-site biological treatment methods has been observed to be effective in addressing contamination issues in soil and water media (Katsoyiannis and Zouboulis 2004). The recent focus of the research community has been directed toward the heightened interest in the biological removal of (HMs) from water (Razzak et al. 2017). The action of mobilization or the ability to immobilize in HMs is essential for aiding microbial cleanup. This is subsequently followed by a series of intricate biochemical reactions, including biomethylation, oxidation, chelation, reduction, and modifying the metallic complex (Pratush et al. 2018). Higher-oxidation state metals are solubilized by microbes into lower-oxidation state metals by enzymatic catalysis (Cumberland et al. 2016). Microorganisms transfer HMs and transform them into non-risk structures by means of membrane-linked transport pathways (Igiri et al. 2018). Microbial entities employ mechanisms like bioleaching, biotransformation, bioaccumulation, and biosorption to sustain their existence within an environment contaminated with metallic substances. Thus, the subsequent mechanisms are employed for microbial bioremediation: The process of eliminating harmful metals involves various mechanisms employed by microorganisms. These include the utilization of cell wall components and specialized proteins and peptides like metallothioneins (MTs) and phytochelatins. Additionally, microorganisms produce compounds such as bacterial siderophores, which are predominantly catecholate, unlike fungi that generate hydroxamate siderophores. Another strategy is to modify biochemical pathways to prevent the absorption of metals. Enzymes are also employed to convert metals into harmless forms. Lastly, microorganisms employ low-efflux systems to decrease the concentration of metals within their cells (Jan et al. 2014).

### Biosorption

It is worth mentioning that Rushhoft (1949) made a significant finding regarding the adsorption effect of microorganisms on heavy metal ions in wastewater. In the 1950s, researchers with a deep understanding of microbiology made an intriguing discovery: a certain strain of bacteria with a fascinating flocculating effect. In 1989, researchers discovered the potential of the biosorption method in addressing cruise oil spill pollution. They also found that this method could be highly effective in removing heavy metals using biological adsorbents (Luo et al. 2022). Microorganisms use their cellular structure to absorb HM ions, which they then sorb onto the binding sites of the cell wall in a process known as biosorption (Malik 2004). The observed phenomenon can be characterized as a passive uptake mechanism that operates independently of the metabolic cycle. Biosorption is an innovative technique for removing HMs from drainage. This method is deemed an effective detoxification operation in the removing of HMs until extremely minor concentrations. Biosorption, a crucial initial process in the elimination of toxic metals by microorganisms, is contingent upon pH and involves the modulation of the isoelectric point within a solution. This modulation subsequently impacts the overall negative charge and causes changes in the ligands’ ionic state, like (COOH) group residues, (P=O) group residues, S–H groups, and (–NH_2_) acid groups (Saǧ et al. 1995). Biosorption is a type of adsorption operation which contains rigid state which is known as sorbent and fluid state which is known as solvent. Lifeless matter does not need developed medium for its evolution which is the primary benefit of utilized lifeless matter over viable matter. In this manner, potential sorbents, such as sawdust, yeast, bacteria, fungi, and algae, are utilized due to of their great sufficiency and cheap in cost (Kaczala et al. 2009). Bio-sorbents are assumed to be very cheap adsorbents, achievable, and obtained from different manufacturing as useless products (Fu and Wang 2011), because the existence of efficacious groups such as ketones, alcohol, carboxylic, aldehydes, ether, and phenolic groups reinforces activity of adsorption which led to great metal elimination (Gadd 2009). The principal agents influencing the potency of bio-bloc in the adsorption operation are metal concentration, pH, dose of the adsorbent, temperature, contact period, etc. (Demirbas 2008). The principal phenomena in the biosorption operation are complexation, ion swap, surface precipitation, and adsorption (Demirbas 2008). Biosorption is a multistep process that includes adsorption, complexation /chelation, exchange of ions, and exterior precipitation (Farooq et al. 2010).

Marine bacteria display remarkable adaptability to changing biological, chemical, and physical conditions, as evidenced by their diverse biochemical pathways and physiological adaptations (De Carvalho and Fernandes 2010). In a manner reminiscent of a microbiologist’s observations, bacteria that come into contact with metals have been found to develop mechanisms to resist their effects (Bruins et al. 2000). Many of these mechanisms involve genes that are found on chromosomes, plasmids, or transposons (Silver and Phung 1996). In a study conducted by Mwandira et al. (2020), the researchers examined the process of biosorption of Pb (II) and Zn (II) using a bacterium *Oceanobacillus profundus* KBZ 3-2. This bacterium was isolated from a contaminated site and has shown tolerance to heavy metals. The results showed that the maximum removal percentage for Pb (II) was 97% at an initial concentration of 50 mg/l, while the maximum removal percentage for Zn (II) was 54% at an initial concentration of 2 mg/l. These results were obtained at a pH of 6 and a temperature of 30 °C. In a study conducted by Das et al. [46], it was found that certain bacterial strains, including *Bacillus coagulans*, *Streptococcus mitis*, *Pseudomonas corrugata*, and *Pseudomonas fluorescens*, exhibited a greater capacity for absorbing Hg. The tested isolates showed a descending order of biosorption for metals as follows: The order of these elements is Hg, Cu, Cr, Cd, Zn, and Co. In a study conducted by Iyer et al. (2005), it was discovered that the exopolysaccharide produced by the marine bacterium *Enterobacter cloacae* exhibited remarkable chelating properties when it came to cadmium (65%), copper (20%), and cobalt (8%) at a heavy metal concentration of 100 mg/l. Marine-derived fungi have the ability to effectively eliminate heavy metal contaminants using both active and passive mechanisms. Fungal biomass, whether living or non-living, has the ability to detoxify and break down dyes found in polluted environments (Dudhagara et al. 2023). In a recent study, Mahmoud et al. (2017) discovered the remarkable potential of heat-inactivated *Aspergillus flavus* strain EGY11 as a bio-sorbent for eliminating harmful heavy metals like Cd(II), Hg(II), and Pb(II) from water solutions. This finding highlights the eco-friendly and highly efficient nature of this strain.

### Bioaccumulation

Bioaccumulation of HMs using microbes is a process known as microbial bioaccumulation or microbial bioremediation. Bacteria and fungus are examples of microbes that can interact with HMs and accumulate them inside of their cells (Nnaji et al. 2023). Bioaccumulation is a phenomenon that occurs when the speed of absorption of a pollutant is above the average at which the pollutant is eliminated from the system. Consequently, the pollutant undergoes entrapment within the organism and exhibits a progressive accumulation phenomenon (Chojnacka 2010). HMs bioaccumulation is the process through which dangerous metals or chemical substances bind inside a cell structure. Metal bioaccumulation is susceptible to the influence of various exposure pathways (such as dietary intake and solution contact) and the geochemical factors that affect the availability of metals in biological systems. The phenomenon of bioaccumulation of metals holds significant value as an indicator of exposure, owing to the fact that metals lack metabolic pathways within biological systems. Bioaccumulation is a dynamic process that encompasses two distinct stages. During the initial phase, metallic ions undergo attachment onto the cellular membrane. During this initial phase, the metabolic activity is observed to be quiescent. Then, metal ions are moved across the cellular membrane. The successful execution of the subsequent phase of this procedure is contingent upon the cells exhibiting a state of heightened metabolic activity. The observed phenomenon of biomass augmentation is contingent upon the diligent preservation of ideal environmental parameters conducive to the proliferation of microorganisms during the subsequent phase. This facilitates the formation of higher quantities of metal ions binding complexes (Zabochnicka-Świątek and Krzywonos 2014).

### Bioleaching

Bioleaching technology is a widely utilized extraction process that has confirms to be highly successful in the elimination of HMs from soil that has been polluted. Bioleaching has become increasingly prevalent in various applications, including extracting the metals from ground ore mining, the cleanup of HMs-contaminated land, the elimination of HMs from sludge, the extraction of manganese from electrolytic manganese slag, and the recovery of valuable metals from old batteries (NareshKumar and Nagendran 2008). When it comes to HMs, bioleaching entails using particular microorganisms, like bacteria or fungus, that have the capacity to oxidize or solubilize the metals. These microorganisms produce and secrete certain chemicals, such as organic acids or enzymes, that facilitate the breakdown and dissolution of the metal-bearing minerals (Sarkodie et al. 2022). In a study conducted by Tian et al. (2022), the bioleaching of rare-earth elements from phosphate rock was examined using *Acidithiobacillus ferrooxidans*. The bioleaching process was discovered to be achieved through bacterial contact and oxidation of Fe^2+^. Based on the findings, it was observed that the phosphate rock had a total leaching rate of 28.46% when the pulp concentration was 1% and the pH was 2. Additionally, the leaching rates of four important rare earths—Y, La, Ce, and Nd—were 35.7%, 37.03%, 27.92%, and 32.53% respectively. Furthermore, the minerals exhibited a smoother and more angular appearance under the scanning electron microscope after bioleaching, suggesting that bacteria played a significant role in the oxidation of Fe^2+^ in the rock. Furthermore, the X-ray diffraction analysis revealed notable changes in the minerals, with a significant decrease in the intensity of the diffraction peaks of dolomite and apatite following microbial activity. This observation provides further evidence of the involvement of extracellular polymeric substances in the bioleaching process. Bioleaching either carried out through direct or indirect approach, in direct approach the organism immediately interact using the metal-containing material, the oxidize the sulfide minerals present in the ore or waste material, releasing metal ions into solution (Rendón-Castrillón et al. 2023). A study conducted by Zhan et al. (2022) examined the use of an indigenous *Acidithiobacillus ferrooxidans* strain to extract Tellurium (Tel) from mine tailings ponds. The results revealed a striking similarity between the bioleaching behavior of Tel and sulfur in sulfide minerals. Specifically, Fe^3+^ initiates the oxidation process of telluride Tel^+2^, transforming it into elemental Tel. Subsequently, bacteria play a crucial role in further oxidizing elemental Tel to Tel^+4^ and Tel^+6^. In addition, the ore sample was examined using a scanning electron microscope and analyzed using Fourier transform infrared spectroscopy. These observations revealed the presence of a bedded structure on the ore’s surface after bioleaching, which acted as a reaction compartment. Furthermore, the changes in active groups suggested a potential attachment between bacteria and the ore. On the other hand, the organism cannot interact with the metal-containing material directly, but act on secondary compounds or by-products generated during mining or metallurgical processes. The acidic conditions created by the microorganisms can then leach out and solubilize HMs from the surrounding materials (Sand et al. 2001).

### Biotransformation

Biotransformation refers to the process by which a chemical compound undergoes structural modifications, resulting in the synthesis of a significantly more polar molecule (Asha and Vidyavathi 2009; Shanu-Wilson et al. 2020). To be clear, when metals and bacteria interact, hazardous metals and organic molecules are changed into forms that are substantially less dangerous. Microbial transformations encompass a range of processes including the synthesis of novel carbon bonds, isomerization, establishing functional groups, oxidation, reduction, condensation, hydrolysis, methylation, and demethylation. Microorganisms like bacteria and fungi have a variety of enzymatic systems that can catalyze the biotransformation of HMs (Pande et al. 2022). The utilization of microorganisms for the purpose of transforming metals has been documented in scientific literature. According to Nagvenkar and Ramaiah (2010), the deadly form of arsenic, As^+3^ can be converted by the microbes *Micrococcus* sp. and *Acinetobacter* sp. into the less harmful form As^+5^, thereby lowering its toxicity. The process of biotransformation of HMs by microorganisms is subject to various factors, which encompass the particular microbial species engaged, environment-related factors like temperature, pH, and food elements availability, and proportion and chemical characteristics of HMs (Igiri et al. 2018). Biotransformation processes have been applied in bioremediation efforts to mitigate the effects of HMs on the environment when converting HMs into less-toxic or more easily removable forms, biotransformation can help reduce their potential harm to ecosystems and human health (Kapahi and Sachdeva 2019).

## Detoxification of heavy metals by microalgae

In the last years, researchers have concentrated on active and inactive algal bio-bloc to remove HMs from wastewater. The capacity for adsorption of living biomass is constrained during the HMs elimination process due to the occurrence of adsorption and uptake operations exclusively during the growth stage. These operations are considered intracellular and involve a more complex adsorption mechanism. Metal adsorption onto the cell wall surface triggers the extracellular process in the context of non-living algal biomass (Lee and Chang 2011).

Various types of microalgae, whether found in freshwater or marine environments, exhibit different levels of tolerance to arsenic, and their growth rates and ability to accumulate arsenic can vary significantly. Studying the bioaccumulation of arsenate by marine microalgae *Phaeodactylum tricornutum*, Morelli and colleagues discovered that the growth rate decreased gradually when the concentration of As(V) exceeded 0.1 µm. The microalgae experienced a significant inhibition of 35% when exposed to 1.0 µm arsenic (Morelli et al. 2005).

The adsorption capabilities of dormant algae can be affected by many environmental conditions, inclusive pH, temperature, and the duration of exposure (Areco et al. 2012). *Lessonia nigrescens* Bory and *Macrocystis integrifolia* Bory, two marine algae species, were used to test the eradication of emerging organic compounds, such as medicines, from a real effluent (Matamoros et al. 2015). Two pilot-scale high-rate algal ponds were used to conduct this experiment. The efficacy of removal exhibited a wide range, spanning from minimal to a maximum of 90%. Notably, the hydraulic retention duration was the sole factor influencing the removal efficiency exclusively during the cold season. The aforementioned phenomenon was not noticed during the period of warmer weather. El-Naggar et al. (2021) revealed that the brown marine alga’s dry biomass *Fucus vesiculosus* exhibited the potential to effectively remove copper ions from aquatic effluents. The findings of this research suggest that this method could be a viable and efficient approach for copper ion removal. The utilization of marine microalgae, specifically *Chlorella vulgaris*, and macroalgae, often known as seaweeds, has witnessed a growing trend in many biotechnological endeavors. One such application is their usage in the bioremediation of wastewater that is contaminated and possesses a low ratio of carbon to nitrogen (C/N) (Sepehri et al. 2020). Algal bio-bloc consists of active effective groups on the wall roof of the cell that reinforce biosorption abilities (Zeraatkar et al. 2016). Ibrahim et al. (2016)’s study looked at the removal of copper, cadmium, chromium, and lead using *U. lactuca* powder (AUP) and activated carbon made from *U. lactuca* (ACU). The use of AUP and ACU allowed for the necessary eliminations to be made at levels of 64.5 and 84.7 mg/g for copper, 62.5 and 84.6 mg/g for cadmium, and 68.9 and 83.3 mg/g for lead, also deduced that the activation of carbon by KOH, which is composed from *U. lactuca,* has more potency in the elimination of HMs than AUP. Desorption of algal bio-bloc can complete utilizing nitric acid, hydraulic acid, and EDTA, which is the algae biosorption operation’s main benefit. Other investigators utilized cyanobacteria jellied settlements separated from rice farms for elimination of Cu, Cd, and Pb from water. They discussed both biosorption and desorption operations in their research (Tran et al. 2016). The recovery of the bio-bloc was made possible using 0.1 M nitric acid and 0.1 M EDTA, which also allowed the adsorbent to be reused. The use of algae in many implementations of the environment was found to have double use in junk water remediation and bio-fuel manufacture (Tran et al. 2016).

## Detoxification of heavy metals by fungi

Fungi have demonstrated significant efficacy in cleanup of HMs because of their inherent ability to adapt and thrive in adverse environments characterized by fluctuating pH levels, severe temperatures, and limited nutrition availability (Deshaware et al. 2021). Chitin, mannan, proteins, glucan, and other polymers with functional groups like (P=O), (COOH), imidazole, and (–OH) are among the ingredients that make up the cellular wall of fungi. Each of these active groups enhance fungi’s ability to carry out adsorption processes (Ramrakhiani et al. 2011). The absorption of HMs by fungi occurs through various mechanisms, including valence transformation, intracellular precipitation, complexation, and ion exchange (Pande et al. 2022). Because of their simplicity and manageability, static systems have been used in fungal cells for metal exploitation in adsorption operations over the past ten years (Rashid et al. 2016). Some of marine fungi which used for biosorption of different heavy metals are listed in Table 1. Ascomycetes have received much attention in bioremediation of heavy metals in previous studies. Rashid et al. (2016) prepared a bio-composite from dead fungal biomass of *Penicillium fellutanum* and bentonite, and it employed for adsorption of nickel and zinc, where results revealed that promising adsorption and recycling potential. Other study examined lifeless fungal bio-bloc of four types of marine *Aspergillus* sp. for mercury biosorption and recorded *A. niger* as the perfect effective for mercury bio-sorbent, lifeless bio-bloc of *A. niger* make 40.53 mg/g^−1^ of mercury eliminate under most favorable states (Khambhaty et al. 2008). Assessment of the potential reaction between cell and metal ion revealed the embroilment of (OH) and (NH_2_) groups existing on the surface of cell in mercury biosorption. On the other hand, *A. candidus*, which was isolated from several Indian coastlines, was investigated by Vala (2010) for its ability to withstand and eliminate arsenic (As), where it showed resistance to (As^+3^) and (As^+5^) as 25 and 50 mg l^−1^. The experiment fungus recorded the largest (As) elimination (mg/g^−1^) on the third day. Another study looked at the degree of (As) resistance and elimination sufficiency of two tested marine fungi, *A. flavus* and *Rhizopus* sp. based on exposure to 25 mg/l and 50 mg/l of sodium arsenate (As^+3^). The tested fungi represent resistance and (As) bioaccumulation, with *Rhizopus* sp. exhibiting a minimal amount of bioaccumulation (Vala and Sutariya 2012). The strain of *Trichoderma viride* that exhibits tolerance to hexavalent chromium was discovered using water samples from the Mediterranean Sea (El-Kassas and El-Taher 2009). It was possible for this fungus to successfully remove 4.66 mg/g^−1^ of chromium (VI) from the solution. The impact of chromium accumulation on the mycelial and conidial structures of the fungus was examined using transmission electron microscopy, revealing no observable alterations. Additionally, 13 yeast strains were found in Trench of Japan soil samples collected by Abe et al. (2001). The organism with the highest tolerance for copper has been identified as *Cryptococcus* sp. So, researcher’s remanding proposes the function of superoxide dismutase to defeat massive copper stress. *Cryptococcus* sp. yeast separated from deep sea, when multiplication in existence of several proportions of HMs salts as cadmium chloride, copper sulfate, lead acetate, and zinc sulfate, showed notable multiplication in existence of 100 mg l^−1^ metal proportion. Comparatively more resistance to these metals was demonstrated by the isolated yeast than by other aquatic and terrestrial yeasts. Cell morphological changes were observed due to the presence of HMs. The tested yeast could get rid of 30–90% of the given HMs. The examined *Cryptococcus* sp. was described by the researcher as an ideal candidate for biotreatment of HMs polluted areas (Singh et al. 2013). Furthermore, Oyetibo et al. (2015) evaluated the removal of mercury by surviving and developing cells of Hg-tolerant *Yarrowia* sp. isolated from numerous aquatic environments, such as Hg-contaminated sediments from estuaries. While the growing cells were proposed to fit as an important (Hg) bio-reduction and volatilization factor, the yeast type leftover cells were suggested to be suitable as a reusable bio-adsorbent. In contrast, Taboski et al. (2005) conducted an assessment of the harmful effects of cadmium on two marine fungus species, namely, *Corollospora lacera* and *Monodictys pelagica*, within the aquatic ecosystem. This evaluation involved the examination of their average growth and bio-bloc aggregation. Fungi growth was not significantly affected by the presence of Pb. On the other hand, fungal growth was suppressed as Cd levels rose, notably in the case of *M. pelagica*. The study revealed that a significant proportion, specifically 93%, of lead (Pb) accumulation by *C. lacera* occurred extracellularly, rather than within the fungal cells. *M. Pelagica* exhibited bioaccumulation levels exceeding 60 mg/g^−1^ for cadmium and surpassing 6 mg/g^−1^ for lead. *C. lacera* exhibited bioaccumulation of cadmium levels exceeding 7 mg/g^−1^ and lead levels reaching up to 250 mg/g^−1^. According to Mendoza et al. (2010), there are two marine fungus species, *Dendryphiella salina*, which has the capability to absorb a significant proportion of mercury, ranging from 80 to 92%, from aqueous media. The investigation unveiled the potential utilization of both strains in the process of bioremediation of mercury, particularly through the mechanism of biosorption.

**Table 1:** Biosorption of different HMs using marine fungi

Fungal species	Metal	References
*Aspergillus niger*	Cu Pb Cr(VI)	Dursun et al. (2003)
*Aspergillus niger*	Hg	Khambhaty et al. (2008)
*Botrytis cinerea*	Pb	Akar and Tunali (2005)
*Phanerochaete chrysosporium*	Cu Pb Zn	Iqbal and Edyvean (2004)
*Pleurotus platypus*	Ag	Das et al. (2010)
*Rhizopus oryzae*	Cu	Fu and Viraraghavan (2001)
*Trichoderma viride*	Cr(VI)	El-Kassas and El-Taher (2009)
*Corollospora lacera* and *Monodictys pelagica*	Pb Cd	Taboski et al. (2005)
*Dendryphiella salina*	Hg	Mendoza et al. (2010)
*A. flavus* and *Rhizopus* sp.	As	Vala and Sutariya (2012)
*Cryptococcus* sp.	Cd Cu Pb Zn	Singh et al. (2013)

**Fig. 4:**
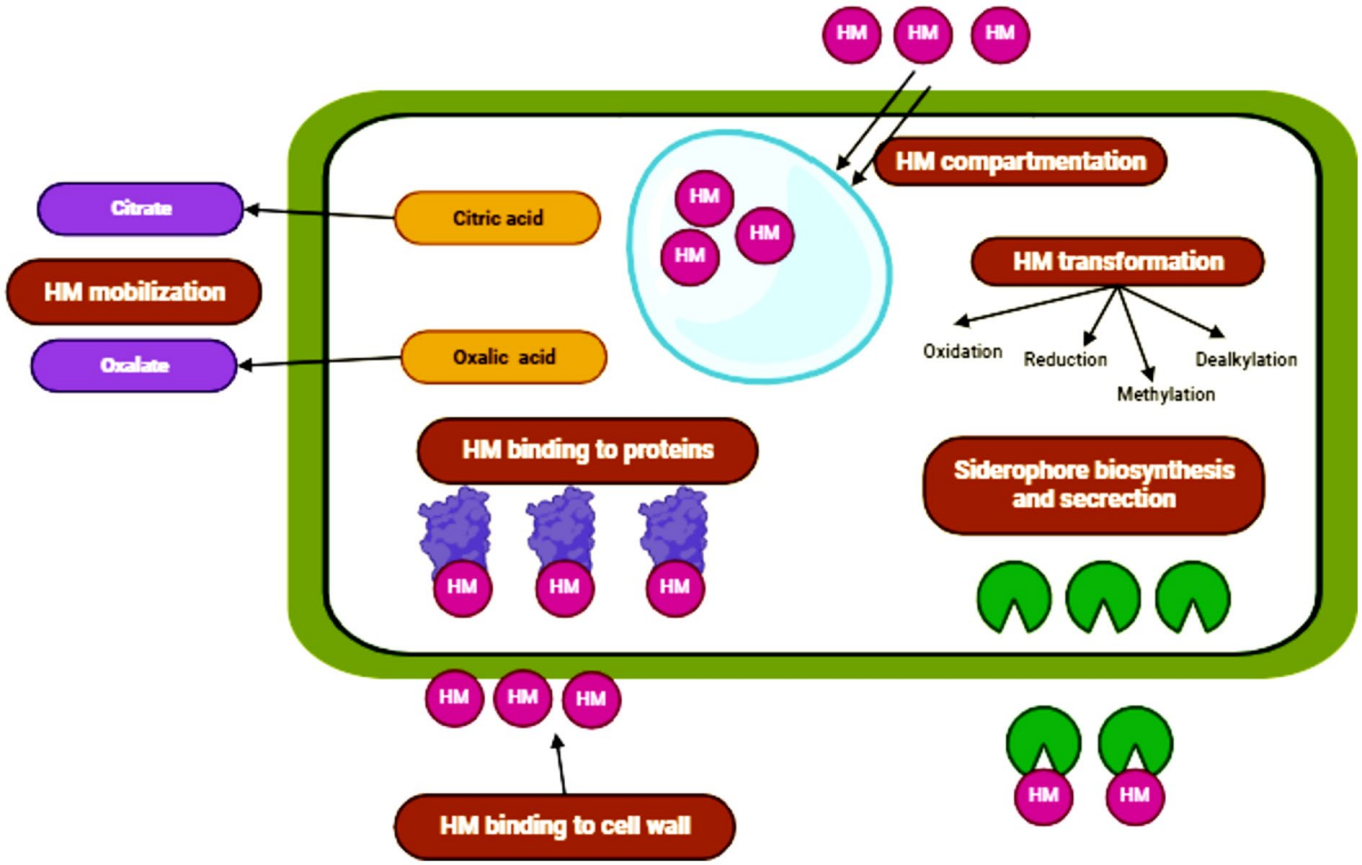
The putative detoxification mechanisms for HMs in fungus

In a study conducted by Gomathi et al. (2012), the efficacy of chromium removal was investigated three types of Thraustochytrids, namely, *Aplanochytrium* sp., *Thraustochytrium* sp., and *Schizochytrium* sp. comprehensive investigations, encompassing adsorption kinetics and optimization techniques, were conducted utilizing *Aplanochytrium* sp. as the subject of study. The results demonstrated that the test fungus exhibited a remarkable chromium removal efficiency of 69.4%. According to Siddiquee et al. (2015) the potential pathways involved in the detoxification of HMs have been divided into two steps. The first step entails the expulsion of chemical compounds into the extracellular environment or cell wall, which later attach to metals, making them physiologically inaccessible and minimizing their potential to injure cells. When the harmful substances enter the cellular structure, the second step is seen. In this procedure, poisonous metal ions are chelated within the cytosol, which results in the deactivation and sequestration of dangerous metals away from sensitive metabolic pathways (Avery et al. 1992). Therefore, the cellular sequestration of metals, metal adhesion to cell membranes, chemical transformations, and the immobilization of metal inside cells are the cellular defensive mechanisms used by fungi and oomycetes to combat excessive quantities of toxic metals in the environment (Fig. 4). In a study conducted by Gao et al. (2022), it was found that soil in mining areas became polluted with heavy metals. This pollution had a direct impact on the physico-chemical properties of the soil, which in turn affected the structure and distribution of microbial communities in the area. The microbial community structure in mining areas is primarily influenced by soil pH. However, it had a beneficial impact on the fungal community and a detrimental impact on the bacterial community. Findings suggest that various microorganisms in the vicinity of the tailings ponds exhibit distinct reactions to environmental factors. In addition, there was a negative correlation observed between the Actinobacteriota and heavy metals such as Zn, Cu, Cd, and Pb. El-Kassas and El-Taher (2009) identified several important factors that have been demonstrated to be helpful in enhancing the elimination of Cr (VI). These variables included the pH of the solution, the ratio of metal to bio-sorbent, the length of time the bio-sorbent was in contact with the metal, and the makeup of the bio-sorbent.

## Detoxification of heavy metals by bacteria

**Fig. 5:**
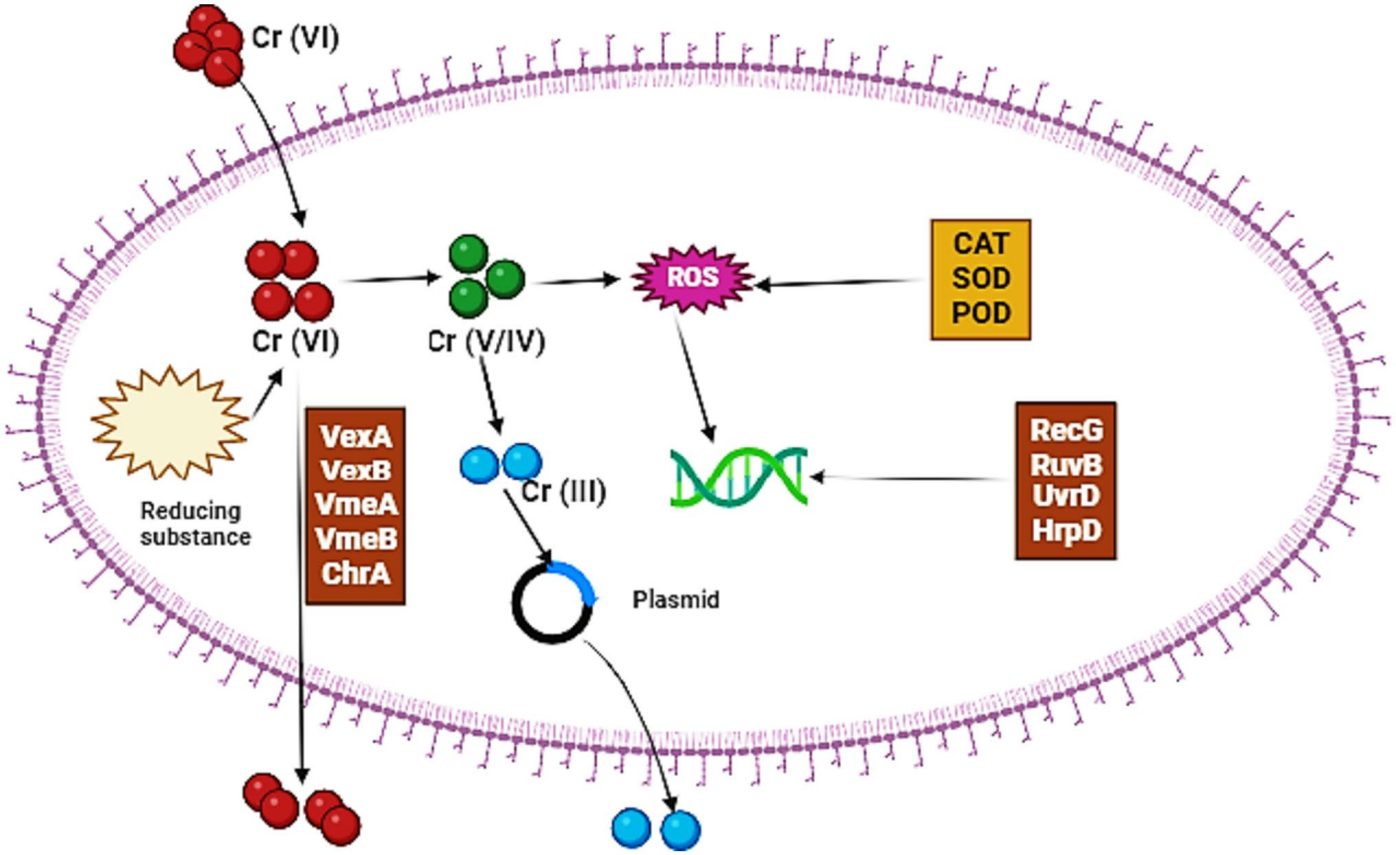
Bioremediation of heavy metal (Cr VI) using bacteria

The majority of bacteria found in seawater belong to the viable but unculturable group. In order to address this issue and explore the variety of bacterial species in marine environments, numerous sophisticated methods have been employed. These include metagenomics, amplification of the 16S rRNA gene, and denaturing gradient gel electrophoresis (Hussain and Khan 2020), cloning, and restriction fragment length polymorphism have been used (Liu et al. 1997). Numerous characteristics of bacteria, such as their availability, small size, and flexibility, excite scientists to concentrate on the bio-adsorbent of bacteria for the elimination of (HM_S_) from junk water. Numerous active groups, including (R-CHO) aldehydes, (–C=O–) ketones, and carboxyl (–COOH) groups, are present in the bacteria’s cell wall. Bacterial bio-bloc is frequently used in the shape of engaging substance to adsorbent for the elimination of HMs from the liquid solutions (Qu et al. 2014). At the three depths sampled, there were significant amounts of bacteria from the Bacteroidetes and Verrucomicrobia phyla, along with numerous unclassifiable bacteria genera. A significant portion of the sequences in our samples belonged to the *Alphaproteobacteria* and *Gammaproteobacteria* classes, accounting for around 24%. The bottom samples had a higher abundance of *Gammaproteobacteria*, making up around 31% of the total community. On the other hand, the surface samples had a higher abundance of *Alphaproteobacteria*, accounting for approximately 23% of the total community. The microbial community structure at the three depths was significantly influenced by temperature and phosphate. The correlation between the separation of bacteria in surface and bottom samples and temperature was quite significant (*r* = 0.72). It was found that temperature had the greatest statistical impact on the differences observed in the microbial community structure (McFarlin et al. 2017). Mohapatra et al. (2017) reported the bioremediation of several harmful metals in salty environments using salt-tolerant bacteria derived from various marine habitats. However, *Pseudomonas* sp. was isolated and characterized by Safahieh et al. (2012) from sediments in the Persian Gulf; this specific strain showed resistance to HMs and polyaromatic hydrocarbons (PAHs). Camacho-Chab et al. (2018) used the biopolymers *Microbacterium* sp. (MC3B-10) and *Bacillus sp.* (MC3B-22) to calculate the biosorption capacity of cadmium. Marine bacteria, notably *Microbacterium* sp. (MC3B-10) and *Bacillus* sp. (MC3B-22), produced these biopolymers. DNA sequencing was used to identify these bacteria, and the results showed a 99.9% similarity to *B. firmus*. So, the *Microbacterium* sp. (MC3B-10) and *Bacillus firmus* (Extracellular Polymeric Substance) demonstrated maximal sorption capacities of 97 mg/g^−1^ and 141 mg/g^−1^ for Cd^+2^ ions, respectively, under circumstances of pH 7 and a temperature of 28 °C. Otherwise, a batch system for the simultaneous biosorption and bioaccumulation of chromium was created, using tea waste bio-bloc as an aid material for the engagement of bacterial biofilm, specifically *E. coli* (Gupta and Balomajumder 2015). The higher (6.329 mg/g) level of (Cr) metal utilization using bio-sorbent was observed. The bio-adsorption method for the reduction process of (Cr) in the biosorption of *E. coli* was defined using the Freundlich isotherm model. The same researchers conducted the same type of experiment, a study using *Bacillus* sp. as an aid material in tea waste bio-bloc for the reduction of (Cr). The second-order pseudo-model perfectly explained the reduction of (Cr) using bio-sorbent, and the higher better exploit of (Cr) using bio-sorbent appeared as (741.389 mg/g) (Gupta and Balomajumder 2015). There is another research in which they utilized bacterial bio-bloc itself in the metal elimination operation (Lakshmanan et al. 2010), which used lifeless and life viscous Arthrobacter bio-bloc for the decreasing of poison Cr^+6^ to the minimal-poison Cr^+3^ at pH from 1 to 2. These results demonstrated that living biomass has a perfect potential for the reduction of Cr^+6^, as predicted by the Langmuir adsorption isotherm model, where the higher value was found to be (1161.3 mg/g). Additionally, some investigations have shown that actinomycetes can function as the ideal bio-sorbent since their cell walls contain active groups (Hlihor et al. 2017). Moreover, Al Turk and Kiki (2011) investigated the removal of HMs from real industrial effluent samples using two isolates of halophilic actinomycetes isolated from saline soil samples obtained from the west of Saudi Arabia. The isolates’ 16S rRNA gene sequences were examined, and they were recognized as *Nocardiopsis halophila* and *Nocardiopsis rosea*, since the removal of Cr (100%) by *N. halophila* and Zn (100%) by *N. rosea*. However, El-Gendy and El-Bondkly (2016) studied two actinomycete strains, *Nocardiopsis* sp. (MORSY1948) and *Nocardia* sp. (MORSY2014), to be powerful active bio-sorbents from contaminated areas. Interestingly, these findings demonstrated that HMs reduction was more effective with dead biomass than with live cells. Both strains can be employed to extract harmful HMs from wastewater, and under ideal conditions, this removal process was observed to approach 100% efficiency in aqueous solutions when the dosage of the sorbent was increased to 0.4%. A marine *Nocardiopsis* sp. that was separated off the west coast of India was cultivated on a variety of carbon and nitrogen sources, with olive oil and ammonium chloride at a C/N ratio of 2:1 yielding the greater production (Khopade et al. 2012). In a recent study by Staicu et al. (2022), it was found that under anoxic conditions, the bioremediation of a retentate contaminated with metals and dominated by arsenic showed promising results. The use of *Shewanella* sp. O23S led to significant removal yields for various elements, including arsenic (8%), cobalt (11%), molybdenum (3%), selenium (62%), antimony (30%), and zinc (40%). When 1 mmol l^−1^ cysteine was added, the removal rate of certain elements saw a significant increase. For instance, the removal rates for elements such as As, Co, Mo, Se, Sb, and Zn were boosted to 27%, 80%, 78%, 88%, 83%, and 90%, respectively. Moreover, the efficiency of *A. tucumanensis* bio-emulsifiers for the retrieval of (Cu) and (Cr) from polluted grounds by using rinsing technique at a lab was examined. The researchers revealed that the bio-molecules were capable of mediating (Cr) recovery, with double the elimination proportion comparison to that appear when utilizing de-ionized water. But they were not efficient in eliminating (Cu) from the ground. The Streptomyces separated from India shore was examined and defined as a perfect bio-surfactant maker by traditional examining process (drop collapsing, hemolytic vitality, and lipase construction) besides its HMs tolerance efficiency (Lakshmipathy et al. 2010). Moreover, Dash and Das (2014) identified two highly Hg-resistant isolates, *Bacillus thuringiensis* (from marine environment) and *Bacillus* sp. (from metal manufacturing waste) and noted that the marine Bacillus isolate represented more Hg volatilization adequacy. They proposed that bacteria from marine origin could be a good choice for reinforced bioremediation of (Hg) polluted area. According to De et al. (2014), *B. cereus,* a mercury-resistant and marine biofilm-forming bacterium, has been identified as a promising candidate for the cleanup of mercury-contaminated waste. Extracellular polysaccharides, extracellular enzymes, and bio-surfactants were discovered to be related to the improved bioremediation capacity of biofilm-forming bacteria. Also, Mulik and Bhadekar (2017) conducted a screening of bacteria from the Antarctic seawater area, isolated in order to assess their tolerance toward HMs and their efficacy in removing these metals from the environment. The test isolates showed tolerance to lead, nickel, cadmium, and chromium at concentrations between 300 and 600 ppm. The average of the HMs elimination rate was recorded as (6–95.39%). *Halomonas* sp. removed cadmium (88.2%), while *Kocuria* sp. demonstrated the elimination of cadmium (95.4%) and lead (86.7%). In the *Kocuria* sp. investigation, it was found that *Halomonas* sp. removed 20.29% of nickel through cell bio-adsorption, while 85% of cadmium was found to be majorly aggregated inside of *Kocuria* sp. cells. Other observations noted by Acharya et al. (2009) assessed the uranium (U) isolating attributes of a marine, unicellular cyanobacterium *Synechococcus elongatus*, that exists amply in oceans. At a pH of 7.8, it was discovered that this bacterium eliminated (72%) uranium (U). *S. elongatus* was suggested as the ideal choice for uranium (U) bioremediation from ecological aquatic systems because it can remove uranium (U) using living/ dead cells or extracellular polysaccharides produced by the bacterium. In their investigation, Henson et al. (2015) discovered a strain of *Microbacterium* sp. (Cr-K29) that demonstrated a remarkable capacity to remove 88% of the harmful metal chromium (VI) from water that had been contaminated with this substance. According to Pattanapipitpaisal et al. (2001) when used in an immobilized microbial condition on a solid substrate, *Microbacterium liquefaciens* has the capacity to remove about 81% of chromium (VI) from wastewater. Bacteria use a variety of techniques to aid in the removal of HMs. Bacterial species can use HMs ions in their metabolic operations or, alternatively, they can neutralize HMs ions using soluble enzymes produced inside the bacterial cells (Ahemad 2015). Figure 5 illustrates the entire process of bioremediation of Cr (VI) by bacteria. Moreover, Table 2 illustrates bioremediation of different heavy metals using different bacterial strains.

**Table 2:** Helpful bacterial strains for heavy metals biotreatment separated from aquatic sources

Metal bacterial strain	Isolation sample	Heavy metals recovery	References
As^+5^ *Streptomyces* sp. VITDDK (3)	Samples of marine soil taken in the Ennore saltpan	98%	Deepika and Kannabiran (2010)
Cd^+2^ *Streptomyces orientalis* VITDDK (1) and *Streptomyces aureomonopodiales* VITDDK (2)	At the Ennore saltpan, samples of marine soil were taken	60% and 70%	Lakshmipathy et al. (2010)
Cd^+2^ *Streptomyces* sp. R (22) and R (25)	samples of sediment from the Sal River near Tucumán, Argentina	98%	Amoroso et al. (2000)
Cr^+6^ and Cr^+3^ *Streptomyces* sp. MS (2)	Egyptian marine sediment	75 mg l^−1^	Mabrouk (2008)
Cr^+3^ *Streptomyces* sp. VITSVK (9)	Indian Bengal, marine sediment	2200 mg l^−1^	Saurav and Kannabiran (2011)
Cr^+6^ *Streptomyces* sp. VITSVK (9)	Indian Bengal, marine sediment	1400 mg l^−1^	Saurav and Kannabiran (2011)
Cr^+6^ *Streptomyces* sp. RSF (17) and CRF (14)	Pakistan, Punjab, near of saline farmlands	90%	Javaid and Sultan (2013)
Cr^+6^ *Streptomyces violaceoruber* LZ (26–1)	Gansu province, China’s Lanzhou stretches of the Yellow River	92.86%	Chen et al. (2014)
Cu^+2^ *Streptomyces* sp. A (160)	India’s Bay of Bengal	480 mg l^−1^	Yadav et al. (2009)
Zn^+2^ *Streptomyces variabilis* (NGP)	Indian marine sediments	66.47%	Selvam and Vishnupriya (2013)
Cu^+2^ *Streptomyces albogriseolus* (NGP)	Indian marine sediments	57.35%	Selvam and Vishnupriya (2013)
U^+6^ *Synechococcus elongatus* (DU/75042)	The ocean, India	72%	Acharya et al. (2009)

## Factors affecting heavy metals bioremediation

Numerous factors, both living and non-living, have a significant impact on the behavior and development of microbial cells, consequently influencing a wide range of biological processes within a microbial community. The bioremediation process is influenced by its complex and diverse surroundings. Microorganisms possess a remarkable capacity to adjust to their surroundings, despite some constraints. Environmental factors such as temperature, pH, low-molecular-weight organic acids, and humic acids have the potential to impact the way heavy metals are transformed, transported, and their valence state altered. Additionally, these factors can influence the bioavailability of heavy metals to microorganisms.

### Physiochemical factors affecting bioremediation

Physiochemical factors encompass a range of parameters that play a crucial role in microbial environments. These parameters include redox potential (Eh), pH levels, ionic strength, solubility, the presence or absence of electron acceptors and donors, temperature, and the age of organometallic ions. Microbes play a crucial role in removing toxic metals through a process called biosorption. This process is influenced by the pH value, which affects the isoelectric point in a solution. As a result, the net negative charge and the ionic state of ligands, such as carboxyl residue, phosphoryl residues, S–H groups, and amino acid groups, undergo changes [36]. The solubility of metal ions is influenced by pH values, with higher solubility observed at lower pH levels. This, in turn, impacts the adsorption process on the surface of microbial cells (Han and Gu 2010). Toxic metal ions, such as Zn^2+^, can significantly impede the process of bioremediation by acting as a respiratory inhibitor in microbes. The biodegradation processes are influenced by the presence of electron acceptors. For instance, aerobic microbes rely on oxygen, while anaerobic microbes utilize NO_3_^1−^, SO_4_^2−^, and Fe (III) oxides (Lovley 2003).

### Biological factors affecting bioremediation processes

The significance of biological factors may not be immediately apparent, but their importance becomes evident when implementing a bioremediation technique. Microbes possess certain inherent characteristics that impact substrate degradation. One such characteristic is the presence of plasmid-encoded genes, which confer specificity for substrates and encode specific enzymes. Interestingly, in nature, bacterial cells have demonstrated a wide range of substrate specificities (Toyama et al. 2011).

### Climate change and bioremediation process

Factors such as increased levels of CO_2_ and rising atmospheric temperatures are indicative of the global climate change. The soil microbial community is of utmost importance in the carbon cycling process, as it has been observed that microbes possess a heightened ability to decompose soil organic matter when exposed to higher levels of CO_2_ (Sowerby et al. 2005).

## Recent technologies in heavy metals bioremediation

### Green bio-adsorbents

Microbial extracellular polymer substance (MEPS) is a cost-effective, environmentally friendly, and biodegradable substance that greatly enhances the ability of microorganisms to adsorb heavy metals. This enables the safe and pollution-free disposal of wastewater. Therefore, it has garnered significant interest (Pi et al. 2021; Yu et al. 2020). In addition, MEPS has the potential to minimize the harmful impact of heavy metals on cells and shows promise as a bio-sorbent for heavy metals. Studies have indicated that the role of MEPS in metal ion adsorption surpasses that of the cell. Through their study of *Desulfovibrio vulgaris*, scientists discovered that the ability of biological cells to adsorb Ca^2+^ was significantly lower compared to MEPS. In comparison, the adsorption capacity of MEPS for Zn^2+^ and Pb^2+^ surpasses that of microorganisms (Lu et al. 2021). The adsorption mechanism of MEPS for heavy metals involves various processes such as surface complexation, ion exchange, redox reactions, electrostatic adsorption, and inorganic microprecipitation (Ledin 2000; Priyadarshanee and Das 2021). Modern technology like genetic engineering makes regulating the process of microbial synthesis of extracellular substances possible (Schmid et al. 2015). Researchers have recently conducted a study where they introduced a plasmid with an exo gene into bacteria. The findings of this study revealed that the overexpression of the exo gene led to a significant increase in the production of succinyl chitosan by the bacteria (Jones 2012). Previous studies have shown that the exoA, exoC, exoF, exoP, exoQ, and exoY genes of A. tumefaciens F2 are involved in the production of MEPS. This was determined through experiments involving gene knockout and gene introduction. Notably, when the exoY gene was introduced into the recombinant strain, there was a significant increase in the yield of MEPS. Additionally, the strain exhibited a high adsorption capacity for Ag(I), reaching 85.4 mg g^−1^. Furthermore, Ag(I) was reduced to silver nanoparticles, which is beneficial for the efficient utilization of metal ions (Pi et al. 2020).

### Metagenome sequencing

Shen et al. (2022) conducted a study on the adaptation of microorganisms to sediments contaminated with heavy metals in the western Chaohu Lake. They used metagenome sequencing to analyze the microbial communities in these sediments. A total of 129 phyla, 2631 genera, and 12,989 species were identified in the sediment samples. In the estuary of Nanfeihe River (ENR), the bacterial biomass made up 22.84% of the total numbers, while the viral quantity accounted for 70.69%. Additionally, the microbial community compositions in ENR differed from those found in other locations. The metagenomics sequencing and functional gene annotation revealed that the ENR harbored several functional genes associated with nucleic acid transport and metabolism, ribosome architectures and biological origin, replication recombining and repair, as well as inorganic ion transport and metabolism. An investigation of the Kyoto Encyclopedia of Genes and Genomes indicated that the sediments from ENR included a high concentration of enzymes associated with the transportation and reduction of heavy metals.

## Limitation of bioremediation using marine microorganisms

Bioremediation is accompanied by numerous advantages, but it also entails certain disadvantages. Optimal environmental conditions must be controlled to promote microbial growth and accelerate the rate of degradation, in order for the procedure to be effective. Additionally, certain chemicals, such as chlorinated organic contaminants, high aromatic hydrocarbons, and radionuclides, exhibit resistance to microbial action. The duration of the process is relatively lengthy, and it is not always possible to consistently get the desired residual levels of pollutants. Proficiency and extensive knowledge are essential for the execution of this technique. Occasionally, it is necessary to conduct small-scale laboratory experiments prior to implementing a project in the field. The constraints of phytoremediation encompass extended temporal durations, the concentration and bioavailability of pollutants or contaminants to plants, the toxic impact of pollutants on plants, and the incapacity to decompose organic contaminants due to the absence of specialized degradative enzymes.

Depending on the particular location, certain pollutants may not undergo complete conversion into benign substances. If the transformation process halts at an intermediate product, it is possible that the intermediate compound may exhibit higher toxicity and/or mobility compared to the parent compound. Additionally, many stubborn pollutants cannot undergo biodegradation. Improper application of injection wells can lead to blockage caused by excessive microbiological growth resulting from the introduction of nutrients, electron donors, and electron acceptors. The presence of high levels of heavy metals and organic compounds hinders the functioning of native microorganisms. Acclimatization of microorganisms is typically necessary for in situ bioremediation; however, this process may not occur for spills and stubborn substances.

## Conclusion and future perspectives

Despite the advancements made in HMs bioremediation by marine organisms like algae, bacteria, and fungi, the majority of the research on this topic is limited to laboratory-scale studies. Nevertheless, the bioremediation potential of bacteria, fungi, and algae from marine or aquatic sources displayed in the current work shall be compared to favorable circumstances to evaluate the quality or efficiency of the operation. There is still a shortage of scientific information on bioremediation methods that may effectively clean up HMs polluted soil, slurries, and aquatic systems on a large scale. However, transferring methods and techniques from basic research into practical science and optimizing in situ remediation employing marine bacteria, fungi, and algae will not be feasible unless a profound knowledge is attained. Nowadays, there are increased risks to the environment and public health due to the HMs intake from wastewater. In order to meet the environmental regulations proposed, various biological methods like bioaccumulation, bioleaching, biotransformation, and biosorption for the bioelimination of HMs were developed. This review discussed the usable biotreatment technologies for the removal of HMs using marine microorganisms. It also highlighted the bioremediation advantages and limitations of biotreatment methods to find out the assuring applicable technique for HMs removal from aquatic environment in large scale. Microorganisms are widely distributed and exhibit rapid growth, adapting to differing levels of various hazardous metal ions. The use of genetically modified microorganisms (GEMs) has enhanced the effectiveness of microbial remediation. However, its practical implementation raises problems regarding legality, ethics, and biosafety. Current endeavors are being made to attain a more comprehensive comprehension of the process by which metals are mobilized, absorbed, transported, and accumulated within plants at a molecular level. Undoubtedly, the understanding of molecular science and the application of nanotechnology have significantly contributed to the investigation of novel approaches for the restoration of places contaminated with heavy metals. However, further study is required to discover novel approaches for the removal of heavy metals, specifically addressing concerns regarding biosafety, emerging contaminants, and the effectiveness of genetically modified microorganisms and transgenic plants. Further investigation is necessary to employ an experimental methodology for gathering data from several disciplines and employing mathematical modeling to enhance predictive accuracy. In order to enhance the environmental applicability, it is necessary to incorporate the collected experimental data into several methodologies to evaluate the efficacy of bioremediation.

## Data Availability

Data will be made available on request.
